# The Mechanisms of the Regulation of Immune Response in Patients with Comorbidity of Chronic Obstructive Pulmonary Disease and Asthma

**DOI:** 10.1155/2016/4503267

**Published:** 2016-08-31

**Authors:** Elena P. Kalinina, Yulia K. Denisenko, Tatyana I. Vitkina, Elena G. Lobanova, Tatyana P. Novgorodtseva, Marina V. Antonyuk, Tatyana A. Gvozdenko, Vera V. Knyshova, Anna V. Nazarenko

**Affiliations:** Institute of Medical Climatology and Rehabilitation Treatment, Vladivostok Branch of Far Eastern Scientific Center of Physiology and Pathology of Respiration, 73g Russkaya St., Vladivostok 690105, Russia

## Abstract

*Background*. Comorbidity of chronic obstructive pulmonary disease (COPD) and asthma (asthma COPD overlap syndrome, ACOS) is a significant problem in pulmonary practice, whose pathogenetic issues are not clarified yet.* Objective*. To study the features of the regulation of immune response in patients with comorbid COPD and asthma.* Methods*. We assessed the levels of CD3^+^, CD4^+^, CD8^+^, CD4^+^/CD8^+^, CD19^+^, CD25^+^, HLA-DR, total IgE, TNF-*α*, IL-4, IFN-*γ*, TXB_2_, and LTB_4_ in patients with comorbid COPD and asthma.* Results*. The study involved 44 people with COPD, 39 people with asthma, and 12 people with comorbid COPD and asthma. The specific features in comorbid COPD and asthma were lymphocytosis, increased absolute count of T-helper cells, increased cytotoxic T-lymphocytes in relative and absolute count, increased relative and absolute numbers of B-lymphocytes, and high levels of total IgE. The elevated levels of TNF-*α* and IL-4 and inhibition of IFN-*γ* production were detected. The content of LTB_4_ was maximal; TXB_2_ levels were higher than in control group but lower than in COPD and asthma.* Conclusion*. In comorbid COPD and asthma inflammation increased even during stable period. High levels of eicosanoids, low production of Th1-type cytokines, and active synthesis of opposition IL-4, along with increased IgE, indicate the activation of Th2-type immune response.

## 1. Introduction

Comorbidity of chronic obstructive pulmonary disease (COPD) and asthma (asthma COPD overlap syndrome, ACOS) is one of the most important problems in current pulmonary practice, but many pathogenetic, diagnostic, and therapeutic aspects of the problem remain unsolved yet [1, 2]. The basis of COPD and asthma is the chronic inflammation of the bronchial tree, which is impairing the integrity of the epithelial layer and causing bronchoconstriction. However, the nature and mechanisms of inflammation in COPD and asthma-caused obstruction differ noticeably. Despite the variety of clinical symptoms of these diseases the development of inflammation is largely determined by immune system [3–6]. It is known that eosinophilic inflammation in asthma is controlled by CD4^+^ lymphocytes [7, 8]. The disorders of respiratory function are occasional and reversible. In COPD inflammation involves neutrophils, macrophages, and CD8^+^ lymphocytes. An important feature of COPD is a progressing pulmonary obstruction concurrent with airway remodeling [8, 9]. Despite the differences in the development mechanisms of asthma and COPD, these diseases have some common features in clinical manifestations and prevention and treatment strategies [10].

Comprehending the mechanisms of ACOS, there must be considered that the stacking of pathogenetic processes and the additional mutual burdening are highly possible. Currently, only the first attempts to solving the issue are taken. The problem of the clinical and laboratory features of the diseases in the pathogenic and applied diagnostic aspects remains unresolved too. All of aforementioned questions raise some difficulties in the practical work even for a well-informed specialist.

The aim of the study was to investigate the state of cellular and humoral immunity, the mediators of inflammation in COPD, and asthma and their combination and to establish the features of the regulation of immune response in patients with comorbid course of chronic obstructive pulmonary disease and asthma.

## 2. Materials and Methods

The study involved 115 people (60 men and 55 women). The inclusion criteria were stable COPD (GOLD I or II), mild persistent, controlled, and partly controlled asthma, and ACOS.

During the screening of employees from a certain company COPD GOLD I was firstly diagnosed in 27 people. 17 people had a stable COPD GOLD II, 39 people mild persistent, controlled, and partly controlled asthma, and 12 people comorbid COPD and asthma. The control group consisted of 20 healthy volunteers (6 men and 14 women), nonsmokers and never smoking before, without allergic anamnesis record. The survey of patients was conducted after signing the informed consent and in accordance with the standards of the Declaration of Helsinki (2008).

The diseases were diagnosed by patient medical history, physical examination, measurement of peak expiratory flow (PEF), spirometry with bronchodilator test (spirograph “FUKUDA”, Japan), and laboratory tests. Respiratory failure was diagnosed by clinical symptoms and the levels of venous oxygen saturation (SaO_2_). Criteria for COPD diagnosis were respiratory symptoms (persistent dyspnea increasing during exercise stress, chronic productive cough), presence of risk factors (smoking, occupational factors), family history of COPD, postbronchodilator FEV1/FVC < 0.7, postbronchodilator FEV1 ≥ 80% from normal for COPD GOLD I, and 50% ≤ FEV1 < 80% from normal for COPD GOLD II, in accordance with the Global Strategy for Diagnosis, Management, and Prevention of COPD (GOLD 2016) [11].

Asthma was diagnosed by spirometry tests. Criteria for asthma diagnosis were respiratory symptoms (chronic paroxysmal cough, wheezing, and dyspnea), the presence of allergic reactions and diseases, family history of asthma, normal or >80% from predicted FEV1, postbronchodilator increase in FEV1 > 12% or 200 mL from base level, and average daily PEF variation 3-4% for controlled asthma and 6-7% for partly controlled asthma in accordance with the Global Strategy for Asthma Management and Prevention (GINA 2016) [12].

ACOS was diagnosed in the presence of respiratory symptoms (persistent dyspnea, chronic cough), risk factors (smoking, occupational factors), allergic reactions and diseases before the age of 40 years, family history of asthma and COPD, postbronchodilator FEV1/FVC < 0.7, and increase in FEV1 ≥ 15% or 400 mL from base level.

The survey with validated questionnaires was conducted to assess the symptoms of the basic disease: for evaluation of dyspnea: modified mMRC questionnaire (Modified British Medical Research Council), to assess asthma control level: ACQ-5 test (Asthma Control Questionnaire), and to evaluate the impact of COPD on a patient's daily life and health status: CAT test (COPD assessment test). The criteria for inclusion in the study were the absence of COPD and asthma exacerbations in last two months. The exclusion criteria were the occupational respiratory diseases, cardiovascular diseases (coronary heart disease, hypertension) and their complications, diabetes, thyroid diseases, acute pathological conditions, and exacerbations of chronic diseases. All patients received basic medical treatment depending on the nosology: patients with controlled asthma received beta-agonists with short-term effect according to need; patients with partially controlled asthma, beta-agonists with short-term effect and inhalation corticosteroids (200–400 micrograms per day, 200–500 micrograms in beclomethasone equivalent); and patients with COPD, cholinolytics with long-term effect (18 micrograms per day). ACOS was treated by using cholinolytics with long-term effect (18 mg/day) and inhalation corticosteroids/beta-agonists with long-term effect (200–400 micrograms per day, 200–500 micrograms in beclomethasone equivalent).

Inflammatory activity was evaluated by the state of cell (CD3^+^, CD4^+^, CD8^+^, CD4^+^/CD8^+^, CD19^+^, and CD16^+^56^+^) and humoral (total IgE) immunity. The parameters of cell-mediated immunity were determined by flow cytometry (“BD Multitest 6-Color TBNK” reagent). The total IgE levels in serum were assessed by ELISA (“Xema-Medica” kit). Cytokine profile was evaluated by the levels of pro- and anti-inflammatory cytokines TNF-*α*, IL-4, and IFN-*γ*, determined by flow cytometry (BD Cytometric Bead Array test system, USA) via flow cytometer BD FACSCanto II. FCAP 3.0 software was used for data processing (BD, USA). The secretion of oxylipins was assessed by the levels of their stable metabolites—thromboxane B_2_ (TXB_2_) and leukotriene B_4_ (LTB_4_). For isolation of eicosanoids from serum we used minicolumns (*Minicolumns for Sample Preparation, USA*), and the levels were determined by quantitative ELISA (*Amersham Biosciences UK, Biotrak EIA system*). Measurement was carried out in 96-well plates via spectrophotometer “Biotek Power Wave” (USA). The data were processed in “Statistica” software, version 6.1 (1203C for Windows).

The normality of distribution was tested by Kolmogorov-Smirnov test. The results of statistical processing are presented as mean and standard error of the mean. The results of statistical processing of laboratory tests are presented as median and upper and lower quartiles. To assess differences between the groups in the case of normal distribution we use Student's test and in the case of an abnormal distribution Mann-Whitney test.

## 3. Results

The clinical characteristics of study participants are presented in Table 1. Among patients with COPD (*n* = 44), most were men—32 people (72.7%). COPD GOLD I was diagnosed for 27 people (61.4%) and COPD GOLD II for 17 people (38.6%). Respiratory failure (I and II degrees) was diagnosed in 25 (56.8%) patients with COPD GOLD I and II. Patients had dyspnea during intensive physical activity. Daily physical activity was performed entirely. SaO_2_ level ranged from 92 to 94% for respiratory failure of I degree and from 84 to 89% for respiratory failure of II degree. 28 patients with COPD are smoking (63.6%). The smoking index in patients with COPD GOLD I was 24.4 packs/year and in patients with COPD GOLD II 28.6 packs/year. 10 people with COPD (22.7%) had underweight (BMI < 18.5). The assessment of the life quality by CAT showed a minor impact of COPD on life for majority of examinees. However, in patients with COPD GOLD II the impact on life was 1.9 times greater (*p* < 0.01).

Among patients with asthma (*n* = 39), most were women—23 people (59%). While verifying the diagnosis, controlled asthma was diagnosed for 15 people (38.5%) and partly controlled asthma for 24 people (61.5%). 13 patients with asthma were smoking (33.3%) and their smoking index was 0.35 packs/year. The majority of patients with asthma had overweight, with BMI 28.9 ± 3.24 kg/m^2^. The sensitization to indoor, food, and pollen allergens was noted for 32 people (82%) and hereditary load for 9 people (23.1%). ACQ-5 test showed a controlled course of the disease in only 38.5% of patients.

Among patients with ACOS (*n* = 12) there were 6 men (50%) and 6 women (50%). All patients in this group have COPD GOLD II and mild persistent and partly controlled asthma. Two people were smoking (16.7%), with smoking index 16.3 packs/year. Body weight in the group of patients with comorbidity mainly corresponded to normal values (BMI 18.5–25); for 3 patients an overweight was identified (BMI 27.6 ± 0.3 kg/m^2^). The impact of COPD on health for patients with comorbidity was higher. The average value of SAT in group 4 was significantly higher than that in the group of patients with COPD GOLD I, indicating a moderate impact of disease on quality of life. Dyspnea on mMRC scale was the highest in patients with comorbidity compared with patients with COPD (the minimum level of dyspnea was 1 point and maximum 3 points). The level of control in patients with comorbidity by ACQ-5 test was lower by 31.7% compared with patients with asthma (*p* < 0.01). The sensitization to allergens was noted for 8 people (66.7%), hereditary load was noted for 7 people (58.3%), and asthma episodes before 40 years were noted in 6 patients (50%).

It was revealed that the pronounced changes in the state of cellular immunity are preserved at remission stage of respiratory diseases, in comparison with control group. Comparative analysis of T-cells subpopulations showed that, in group of COPD patients compared with control group, group of patients with asthma and ACOS, the relative (*p*_1-2_ = 0.000001, *p*_2-3_ = 0.000010, and *p*_2-4_ = 0.001279) and the absolute levels of CD3^+^ cells (*p*_1-2_ = 0.000002, *p*_2-3_ = 0.000010, and *p*_2-4_ = 0.000127) were increased (Table 2). The increase in total T-cell population in COPD is due to increase in CD3^+^CD4^+^ lymphocytes. The increase of the absolute number of CD3^+^CD4^+^ lymphocytes in patients with COPD compared with the control group, patients with asthma and ACOS (*p*_1-2_ = 0.000001, *p*_2-3_ = 0.000010, and *p*_2-4_ = 0.0000132, resp.), was shown. The content of lymphocytes with cytotoxic function (CD3^+^CD8^+^) in absolute values was prevalent in COPD patients compared with control group and patients with asthma (*p*_1-2_ = 0.000440, *p*_2-3_ = 0.000010) that affected the immunoregulatory index (CD4^+^/CD8^+^), which was increased to the value of 2.4 standard units (*p*_1-2_ = 0.008355, *p*_2-3_ = 0.000030, and *p*_2-4_ = 0.0000132). The content of B-lymphocytes (CD19^+^) in COPD has a tendency to decrease in comparison with control group. Along with the changes in T-cell immunity the immunoregulatory disorders were also revealed in patients with COPD. The levels of the proinflammatory cytokine TNF-*α* in serum were increased to 48.51 pg/mL and levels of IFN-*γ* to 78.69 pg/mL (*p*_1-2_ = 0.000004). In contrast, the levels of anti-inflammatory cytokine IL-4 were decreased to 43.95 pg/mL in patients with COPD in comparison with other groups (*p*_1-2_ = 0.000034, *p*_2-3_ = 0.000001, and *p*_2-4_ = 0.0000132). The increase in levels of serum LTB_4_ was detected up to 18.00 pg/mL (*p*_1-2_ = 0.000282) and TXB_2_ up to 31.36 pg/mL (*p*_1-2_ = 0.000004) in comparison with control group. Thus, the activation of the immune T-cells, the inhibition of B-cells, the active endogenous interferon synthesis, and high levels of eicosanoids were specific for patients with COPD. The data suggest that the disorders in the immune system are of the Th1 type of immune response, forming the persistent inflammation.

**Figure 1:**
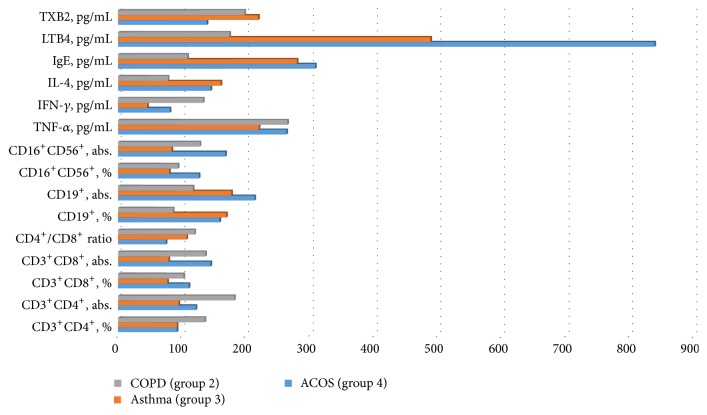
The changes in immune status for patients with COPD, asthma, and ACOS.

Analysis of immunological parameters in patients with asthma revealed the presence of a different kind of disorders in all parts of the immune system (Figure 1, Table 2).

CD3^+^ cells were decreased for all patients with asthma, both percentages (*p*_1-3_ = 0.000150, *p*_2-3_ = 0.000010) and absolute count (*p*_2-3_ = 0.000010, *p*_3-4_ = 0.000160) in comparison with COPD group and ACOS group. The results suggest a persistent inflammation in patients with asthma, even without allergen presence. The absolute numbers of CD3^+^CD4^+^ cells in asthma patients were not significantly different from control group but were lower than in COPD group and ACOS group (*p*_2-3_ = 0.000010, *p*_3-4_ = 0.000165). The absolute numbers of cytotoxic T-lymphocytes (CD3^+^CD8^+^) were decreased in patients with asthma in comparison with COPD and ACOS groups (*p*_2-3_ = 0.000010, *p*_3-4_ = 0.000162). The immunoregulatory index (CD4^+^/CD8^+^) in patients with asthma did not exceed 2.15 standard units. The levels of B-lymphocytes (CD19^+^) in patients with asthma were increased in comparison with control group (*p*_1-3_ = 0.000010) and COPD patients (*p*_2-3_ = 0.000001), indicating the activation of humoral immune system, which is supported by high levels of IgE (*p*_1-3_ = 0.000001, *p*_2-3_ = 0.000010) and is specific for allergic inflammation.

In cytokine profile of asthma patients the high levels of TNF-*α* up to 40.29 pg/mL (*p*_1-3_ = 0.000001, *p*_2-3_ = 0.000154, and *p*_3-4_ = 0.009131) and IL-4 to 90.42 pg/mL (*p*_1-3_ = 0.000010, *p*_2-3_ = 0.000001) were detected, while the levels of IFN-*γ* were decreased to 26.99 pg/mL (*p*_1-3_ = 0.000010, *p*_2-3_ = 0.000001, and *p*_3-4_ = 0.000166). The levels of LTB_4_ in serum were increased to 50.53 pg/mL (*p*_1-3_ = 0.000017, *p*_2-3_ = 0.001055) and TXB_2_ to 34.77 pg/mL (*p*_1-3_ = 0.000001, *p*_2-3_ = 0.000682, and *p*_3-4_ = 0.000509).

The inhibition of T-cell immunity and the production of IFN-*γ* and an increase in levels of vasoactive mediators—eicosanoids (LTB_4_, TXB_2_) and cytokines (TNF-*α*, IL-4)—were observed for asthma in clinical remission, indicating the persistence of the inflammatory process due to the activation of Th2-type immune response.

The study of immunological parameters in ACOS patients has shown a slight lymphocytosis (*p*_1-4_ = 0.000638, *p*_2-4_ = 0.000118, and *p*_3-4_ = 0.12776), the increase in absolute numbers of T-helper cells (*p*_1-4_ = 0.000913, *p*_3-4_ = 0.000165), and relative (*p*_1-4_ = 0.005240, *p*_3-4_ = 0.000150) and absolute (*p*_1-4_ = 0.001686, *p*_3-4_ = 0.000162) content of cytotoxic T-lymphocytes. The levels of B-lymphocytes were also increased, both in relative (*p*_1-4_ = 0.000049, *p*_2-4_ = 0.000128) and absolute (*p*_1-4_ = 0.000028, *p*_2-4_ = 0.000131) count, accompanied by high values of total IgE (*p*_1-4_ = 0.000029, *p*_2-4_ = 0.00012). Along with the changes in cellular immunity the disorders of cytokine profile were detected in patients with ACOS, presented by increased levels of serum TNF-*α* (*p*_1-4_ = 0.000029) and IL-4 (*p*_1-4_ = 0.000029, *p*_3-4_ = 0.009132) and a moderate inhibition of IFN-*γ* production (*p*_1-4_ = 0.000154, *p*_2-4_ = 0.000132). The content of serum LTB_4_ in patients with ACOS had maximum values up to 86.75 pg/mL (*p*_1-4_ = 0.000029, *p*_2-4_ = 0.000132, and *p*_3-4_ = 0.002085). TXB_2_ levels were higher than in control group (*p*_1-4_ = 0.000090) but significantly lower than in patients with COPD (*p*_2-4_ = 0.000132) and asthma (*p*_3-4_ = 0.000509). Thus, the increased activity of the T- and B-cell components of the immune system, high levels of pro- and anti-inflammatory cytokines, and increased levels of LTB_4_ were specific for comorbidity.

## 4. Discussion

The 2-fold increase in content of CD3^+^CD4^+^ lymphocytes and in synthesis of TNF-*α*, IFN-*γ*, and TXB_2_ was revealed for patients with COPD in remission in comparison with other groups of observations, which may indicate the proceeding stimulation of the immune system in response to an antigen.

In patients with asthma in comparison with COPD the absolute numbers of CD19^+^ cells were 1.5-fold increased. The levels of secretion of IL-4 and LTB_4_ were elevated by 2 and 3 times, respectively, compared with the group of COPD. The results suggest the presence of chronic inflammation in patients with asthma, which is supported by the high expression levels of IgE and is characterized by persistent allergic process that leads to a decrease in control over the disease. Several studies have shown that obesity increases the risk of asthma and impairs symptoms control proportionally to the weight gain. Though we found no correlation between the increase in body weight and symptoms of asthma in our study, which is probably due to the fact that patients in our study had overweight without diagnosed obesity.

The obtained clinical and immunological data show that comorbid COPD and asthma are based on increasing inflammation presented even during stable period. Along with almost constant count of total CD3^+^ cell population, the increase in numbers of CD3^+^CD8^+^ lymphocytes was detected in patients with comorbidity, causing severe imbalance in the cell component of the immune system and reducing of immunoregulatory index to 1.5 standard units.

The increase in the levels of B-lymphocytes and high synthesis of IgE, indicating activation of humoral immune system, were detected in patients with ACOS, which is consistent with data from other previous studies [13, 14]. The comparison of CD16^+^CD56^+^ cells returned low values for asthma patients and high for patients with comorbidity, revealing a major role of NK-cells in the pathogenesis of COPD and the formation of mechanisms maintaining chronic inflammation in asthma.

Increasing absolute values of CD16^+^CD56^+^ lymphocytes in patients with comorbidity in comparison with other observed groups indicate the presence of underlying chronic viral and/or bacterial infections, burdening the course of ACOS syndrome and requiring the identification of the antigen and its elimination [15]. The study by Miravitlles et al. [16] suggests the inhalation of corticosteroids and beta-agonists with long-term effect as the most appropriate therapy. Obtained data supports this course of therapy, because the levels of LTB_4_ in serum of patients with comorbid COPD and asthma are 8 times higher than in patients with COPD and 2 times higher than in patients with asthma that confirms allergic hyperreactivity and justifies a good therapeutic effect of aforementioned medications [16].

The important strategic direction in the treatment of ACOS is the determination of the phenotype of the disease by detecting whether prevailing disease is asthma or COPD and revealing the character of inflammation (Th1 or Th2), consistent with the work by Rhee [17].

The allergic sensitization in 66.7% of patients, asthma episodes before 40 years in 50% of patients with ACOS syndrome, high levels of eicosanoids in serum, low production of Th1-type cytokines (IFN-*γ*), and high synthesis of opposition IL-4, together with the increased production of IgE, indicate an active inclusion of the mechanisms of switching to Th2-helpers in patients with ACOS, requiring further studies.

The small sample size is the main limitation of the study. However, a high level of statistical significance of the studied parameters provided an opportunity to highlight the trends in the development of inflammatory processes for comorbidity. A further research with larger sample size for ACOS patients is required to acquire more precise conclusions.

## 5. Conclusion

Thus, the inflammatory processes developing by types 1 and 2 of immune response, which are involving reagins, cytokines, and eicosanoids, turn to the main driving force in the pathological processes in comorbid COPD and asthma (ACOS). Presumably this may be explained by the initiation of systemic damage, by the generalization of the basic mechanisms of inflammation, and by loss of protective role of these mechanisms (the localization of the damage factor) [9, 18]. To summarize, the aforementioned problem requires further thorough diagnostic study with extended immunological study methods for patients with comorbid COPD and asthma.

## Figures and Tables

**Table 1:** The clinical and functional parameters of patients included in study.

Parameters	Control group, *n* = 20 (group 1)	COPD, *n* = 44 (group 2)		Asthma, *n* = 39 (group 3)	Comorbid COPD and asthma (ACOS), *n* = 12 (group 4)
		COPD I (group 2a)	COPD II (group 2b)		
Age, years	32.8 ± 8.32	58.4 ± 2.34	62.7 ± 1.5	42.6 ± 2.38	64.3 ± 1.53
Sex, male/female	6/14	19/8	13/4	16/23	6/6
Smoking status					
(i) Smokers	0	9	14	13	2
(ii) Exsmokers	0	3	2	24	10
Smoking index, pack-year	0	24.4 ± 6.78	28.6 ± 7.87	0.35 ± 0.2	16.3 ± 5.73
BMI, kg/m^2^	26.1 ± 2.21	20.8 ± 1.88	21.7 ± 1.48	28.9 ± 3.24	26.9 ± 2.06
PEF, %	110.77 ± 6.11	97.02 ± 3.43	73.45 ± 2.79 *p*_1-2b_ < 0.001 *p*_2a-2b_ < 0.001	96.76 ± 8.72 *p*_3-2b_ < 0.05	82.5 ± 6.75
Postbronchodilator FEV1, % of the normal	105.1 ± 3.05	96.68 ± 3.98	67.6 ± 2.88 *p*_1-2b_ < 0.01	95.56 ± 7.62	81.72 ± 6.98 *p*_1-4_ < 0.05
Postbronchodilator FEV1/FVC ratio, %	81.56 ± 3.05	60.22 ± 2.18 *p*_1-2a_ < 0.01	56.7 ± 2.47 *p*_1-2b_ < 0.01	74.53 ± 5.33	63.50 ± 4.48 *p*_1-4_ < 0.01
FEV1 increase after inhaling bronchodilator, %	0	9.8 ± 2.34	6.7 ± 2.67	16.2 ± 3.46 *p*_2a-3_ < 0.01 *p*_2b-3_ < 0.01	12.8 ± 2.76
Dyspnea by mMRC scale, score	0	1.0 ± 0.68	1.47 ± 0.1	—	1.5 ± 0.06
COPD influence on patient health by COPD assessment test (CAT), score	0	4.4 ± 0.98	8.4 ± 1.06 *p*_2a-2b_ < 0.01	—	10.3 ± 2.14 *p*_2a-4_ < 0.01
Asthma control by ACQ-5 test, score	0	—	—	0.63 ± 0.02	0.83 ± 0.06 *p*_3-4_ < 0.01

**Table 2:** Parameters of immune status in patients with COPD, asthma, and ACOS.

Parameters	Control, *n* = 20 (group 1)	COPD, *n* = 44 (group 2)	Asthma, *n* = 39 (group 3)	Comorbid COPD and asthma (ACOS), *n* = 12 (group 4)
Leukocytes, 10^9^/L	5.66 (4.75–7.25)	7.90 (7.40–8.30) *p*_1-2_ = 0.000005	5.57 (5.40–5.80) *p*_2-3_ = 0.00001	6.24 (5.90–6.40) *p*_2-4_ = 0.0001

Lymphocytes				
%	31.09 (27.15–32.55)	29.30 (29.00–30.00)	31.92 (30.60–33.10)	36.33 (34.40–38.80) *p*_1-4_ = 0.0006 *p*_2-4_ = 0.0001 *p*_3-4_ = 0.012
Absolute	1727.73 (1331.90–2034.50)	2313.89 (2227.40–2407.00) *p*_1-2_ = 0.0001	1776.05 (1722.60–1820.00) *p*_2-3_ = 0.00001	2269.18 (2029.60–2483.20) *p*_1-4_ = 0.006 *p*_3-4_ = 0.0002

CD3^+^				
%	70.62 (66.71–72.40)	84.62 (80.73–88.83) *p*_1-2_ = 0.000001	63.25 (59.18–67.06) *p*_1-3_ = 0.0001 *p*_2-3_ = 0.00001	70.46 (62.00–79.39) *p*_2-4_ = 0.001
Absolute	1217.72 (960.13–1495.18)	1956.25 (1836.80–2010.58) *p*_1-2_ = 0.000002	1122.72 (1088.11–1189.37) *p*_2-3_ = 0.00001	1584.06 (1539.58–1611.29) p_1-4_ = 0.0009 *p*_2-4_ = 0.001 *p*_3-4_ = 0.0002

CD3^+^CD4^+^				
%	44.47 (41.42–47.31)	60.14 (58.00–61.04) *p*_1-2_ = 0.000001	40.78 (39.80–42.00) *p*_2-3_ = 0.00001	40.81 (39.32–41.41) *p*_2-4_ = 0.0001
Absolute	763.13 (634.98–960.84)	1385.75 (1334.81–1432.19) *p*_1-2_ = 0.000001	723.47 (709.80–736.09) *p*_2-3_ = 0.00001	926.16 (840.46–993.28) *p*_1-4_ = 0.0009 *p*_2-4_ = 0.0001 *p*_3-4_ = 0.0002

CD3^+^CD8^+^				
%	24.65 (21.86–26.01)	25.22 (24.65–26.60)	18.99 (18.58–19.40) *p*_1-3_ = 0.00003 *p*_2-3_ = 0.00001	27.24 (26.20–27.92) *p*_1-4_ = 0.005 *p*_3-4_ = 0.0001
Absolute	426.83 (304.92–502.63)	582.83 (539.84–621.88) *p*_1-2_ = 0.0004	336.99 (327.71–343.53) *p*_2-3_ = 0.00001	617.56 (565.04–673.18) *p*_1-4_ = 0.002 *p*_3-4_ = 0.0001

CD4^+^/CD8^+^ ratio	2.01 (1.44–2.40)	2.40 (2.32–2.46) *p*_1-2_ = 0.008	2.15 (2.11–2.17) *p*_2-3_ = 0.00003	1.50 (1.44–1.51) *p*_2-4_ = 0.0001 *p*_3-4_ = 0.0002

CD19^+^				
%	12.00 (9.22–14.78)	10.31 (9.22–11.10)	20.26 (19.36–20.83) *p*_1-3_ = 0.00001 *p*_2-3_ = 0.000001	19.04 (17.69–20.650) *p*_1-4_ = 0.00005 *p*_2-4_ = 0.0001
Absolute	203.33 (159.45–258.77)	237.87 (212.94–242.70)	359.91 (340.66–385.92) *p*_1-3_ = 0.000001 *p*_2-3_ = 0.00002	433.25 (376.49–496.64) *p*_1-4_ = 0.00003 *p*_2-4_ = 0.0001

CD16^+^CD56^+^				
%	15.09 (10.95–18.27)	14.08 (13.03–15.03)	12.07 (11.00–12.68) *p*_2-3_ = 0.003	19.05 (18.22–20.04) *p*_1-4_ = 0.002 *p*_2-4_ = 0.0002 *p*_3-4_ = 0.0002
Absolute	257.86 (209.69–328.41)	328.75 (301.981–349.13) *p*_1-2_ = 0.007	215.12 (189.73–220.99) *p*_2-3_ = 0.00004	432.41 (394.35–472.85) *p*_1-4_ = 0.00005 *p*_2-4_ = 0.0006 *p*_3-4_ = 0.0002

TNF-*α*, pg/mL	18.32 (13.49–22.11)	48.51 (46.80–50.45) *p*_1-2_ = 0.000004	40.29 (36.80–45.61) *p*_1-3_ = 0.000001 *p*_2-3_ = 0.0002	48.24 (43.78–52.84) *p*_1-4_ = 0.00003

IFN-*γ*, pg/mL	59.17 (54.61–63.45)	78.69 (71.33–86.07) *p*_1-2_ = 0.000004	26.99 (24.70–29.38) *p*_1-3_ = 0.00001 *p*_2-3_ = 0.000001	47.93 (46.13–49.45) *p*_1-4_ = 0.0002 *p*_2-4_ = 0.0001 *p*_3-4_ = 0.0002

IL-4, pg/mL	56.41 (53.34–59.47)	43.95 (41.58–47.09) *p*_1-2_ = 0.00003	90.42 (84.25–94.25) *p*_1-3_ = 0.00001 *p*_2-3_ = 0.000001	81.70 (74.55–87.35) *p*_1-4_ = 0.00003 *p*_2-4_ = 0.0001

IgE, pg/mL	184.75 (160.00–215.00)	200.04 (159.50–233.20)	515.92 (437.00–581.00) *p*_1-3_ = 0.000001 *p*_2-3_ = 0.00001	569.25 (499.40–619.30) *p*_1-4_ = 0.00003 *p*_2-4_ = 0.0001

LTB_4_, pg/mL	10.35 (8.00–12.50)	18.00 (14.00–18.00) *p*_1-2_ = 0.0003	50.53 (37.00–56.00) *p*_1-3_ = 0.00002 *p*_2-3_ = 0.001	86.75 (84.00–89.00) *p*_1-4_ = 0.00003 *p*_2-4_ = 0.0001 *p*_3-4_ = 0.002

ТХВ_2_, pg/mL	15.87 (15.00–17.50)	31.36 (31.00–32.00) *p*_1-2_ = 0.000004	34.77 (34.00–36.00) *p*_1-3_ = 0.000001 *p*_2-3_ = 0.0007	22.00 (19.00–24.50) *p*_1-4_ = 0.00009 *p*_2-4_ = 0.0001 *p*_3-4_ = 0.0005
